# Prevalence and antimicrobial susceptibility of *Arcobacter* species in human stool samples derived from out- and inpatients: the prospective German *Arcobacter* prevalence study Arcopath

**DOI:** 10.1186/s13099-020-00360-x

**Published:** 2020-04-15

**Authors:** Vanessa Brückner, Ulrike Fiebiger, Ralf Ignatius, Johannes Friesen, Martin Eisenblätter, Marlies Höck, Thomas Alter, Stefan Bereswill, Greta Gölz, Markus M. Heimesaat

**Affiliations:** 1 Institute of Food Safety and Food Hygiene, Freie Universität Berlin, Berlin, Germany; 2 Institute of Microbiology, Infectious Diseases and Immunology, Charité– University Medicine Berlin, Corporate Member of Freie Universität Berlin, Humboldt-Universität zu Berlin and Berlin Institute of Health, Berlin, Germany; 3 Labor 28, Berlin, Germany; 4 Synlab MVZ, Berlin, Germany; 5 Labor Limbach, Berlin, Germany

**Keywords:** *Arcobacter*, Humans, Germany, Prevalence, Antimicrobial susceptibility

## Abstract

**Background:**

*Arcobacter* species, particularly *A. butzleri*, but also *A. cryaerophilus* constitute emerging pathogens causing gastroenteritis in humans. However, isolation of *Arcobacter* may often fail during routine diagnostic procedures due to the lack of standard protocols. Furthermore, defined breakpoints for the interpretation of antimicrobial susceptibilities of *Arcobacter* are missing. Hence, reliable epidemiological data of human *Arcobacter* infections are scarce and lacking for Germany. We therefore performed a 13-month prospective *Arcobacter* prevalence study in German patients.

**Results:**

A total of 4636 human stool samples was included and *Arcobacter* spp. were identified from 0.85% of specimens in 3884 outpatients and from 0.40% of specimens in 752 hospitalized patients. Overall, *A.* *butzleri* was the most prevalent species (n = 24; 67%), followed by *A.* *cryaerophilus* (n = 10; 28%) and *A.* *lanthieri* (n = 2; 6%). Whereas *A.* *butzleri, A.* *cryaerophilus* and *A.* *lanthieri* were identified in outpatients, only *A.* *butzleri* could be isolated from samples of hospitalized patients. Antimicrobial susceptibility testing of *Arcobacter* isolates revealed high susceptibilities to ciprofloxacin, whereas bimodal distributions of MICs were observed for azithromycin and ampicillin.

**Conclusions:**

In summary, *Arcobacter* including *A. butzleri*, *A. cryaerophilus* and *A. lanthieri* could be isolated in 0.85% of German outpatients and ciprofloxacin rather than other antibiotics might be appropriate for antibiotic treatment of infections. Further epidemiological studies are needed, however, to provide a sufficient risk assessment of *Arcobacter* infections in humans.

## Background

The genus *Arcobacter* belongs to the family of *Campylobactereaceae* as initially proposed by Vandamme et al. [1]. To date, 29 *Arcobacter* species have been identified [2, 3]. The Gram-negative, motile bacteria are aerotolerant and able to grow at temperatures below 30 °C. *Arcobacter* have been isolated from various sources, such as animals, food products of animal origin, vegetables and environmental waters [4–6]. In animals, *Arcobacter* infections sometimes result in reproductive disorders, mastitis, and diarrhea, whereas the bacteria can also be isolated from healthy carriers [4]. In humans, severe cases following *Arcobacter* infection have been reported including prolonged watery gastroenteritis with abdominal cramps, bacteremia, endocarditis and peritonitis [5, 7, 8]. *A.* *butzleri* followed by *A.* *cryaerophilus* are the predominant species isolated from human specimens, while human infections with *A.* *skirrowii* or *A.* *thereius* have only been rarely reported [9–11]. Nevertheless, the clinical relevance of human *Arcobacter* infections is still under debate. Given that the isolation and identification of *Arcobacter* may fail in routine diagnostic settings, robust epidemiological data on *Arcobacter*-induced morbidities are limited. Thus far, *Arcobacter* prevalences of 0.2–3.6% have been reported for humans [4, 12]. In a recent Belgian study, *Arcobacter* was the fourth most common pathogenic agent in diarrheal outpatients [10]. To date, there are no *Arcobacter* prevalence data for Germany, although since 2002, *Arcobacter* species such as *A.* *butzleri* and *A.* *cryaerophilus* have been classified as serious hazards to human health by the International Commission on Microbiological Specification for Foods [13].

Most infections with *Arcobacter* appear to be self-limiting and do not require antimicrobial treatment; nevertheless, in cases of severe and persistent symptoms antibiotic treatment may be indicated [14]. Several classes of antibiotics, such as fluoroquinolones, tetracyclines and aminoglycosides have been considered for treatment of *Arcobacter* infections [5]. However, a recent meta-regression analysis revealed an emerging resistance of *Arcobacter* species against various antibiotics including fluoroquinolones [15]. Therefore, the objective of the present prospective study was (i) to determine the prevalence of *Arcobacter* spp. in human stool samples in Germany and (ii) to assess the antimicrobial susceptibility patterns of the isolates.

## Results

### Prevalence of *Arcobacter* spp. in human stool samples

In the present study, a total of 4636 human stool samples were included. By using *Arcobacter*-specific isolation procedures, *Arcobacter* spp. were detected in 33 samples (0.85%) obtained from 3884 outpatients and in 3 specimens (0.40%) from 752 hospitalized patients (Table 1). Of the 33 isolates, 21 were identified as *A. butzleri* and 10 as *A. cryaerophilus* by multiplex PCR, while *rpoB* sequencing revealed that two of the putative *A. butzleri* isolates belong to the species *A. lanthieri.* All three *Arcobacter* species were isolated from outpatients samples, whereas only *A.* *butzleri* was isolated from hospitalized specimens. Overall, *A.* *butzleri* was the most prevalent species, followed by *A.* *cryaerophilus* and *A.* *lanthieri*.

**Table 1:** Prevalence of *Arcobacter* spp. in human stool samples collected from October 2017 to October 2018

Patients	No. of samples	*Arcobacter* spp.	Identified species		
			*A. butzleri*	*A. cryaerophilus*	*A. lanthieri*
Outpatient	3884	0.85% [33]	64% (21/33)	30% (10/33)	6% (2/33)
Hospitalized	752	0.40% [3]	100% (3/3)	–	–
Total	4636	0.77% [36]	67% (24/36)	28% (10/36)	6% (2/36)

**Fig. 1:**
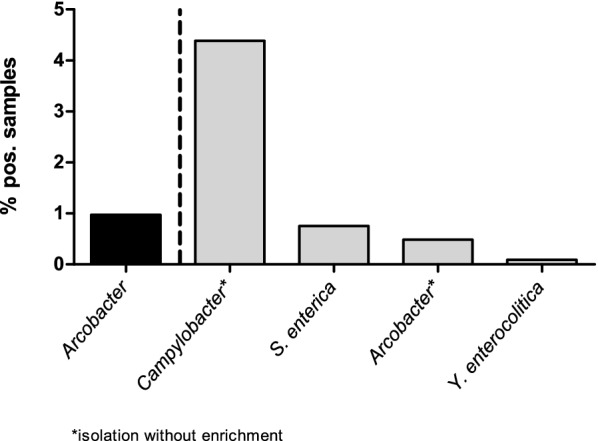
Prevalence of bacterial pathogens in a subgroup of the study population (n = 2257). Black bars: prevalence of *Arcobacter* spp. detected by using *Arcobacter* specific enrichment culture; grey bars: prevalence of bacterial pathogen detected by routine methods

For a subgroup of the outpatients study population (n = 2257), data on other bacterial pathogens were available. While an enrichment step was used for the isolation of *Yersinia* and *Salmonella*, the isolation of *Campylobacter* was performed without enrichment. Within this subgroup *Campylobacter* spp. (4.39%) were the most frequently detected bacterial pathogens, followed by *Salmonella enterica* (0.75%), and *Yersinia enterocolitica* (0.09%) (Fig. 1). Notably in this subgroup, a twofold higher (0.97%) *Arcobacter* prevalence was determined by specific *Arcobacter* enrichment procedures compared to the prevalence determined by the routine diagnostic method for *Campylobacter* without enrichment (0.49%).

### Antimicrobial susceptibilities of *Arcobacter* isolates

**Fig. 2:**
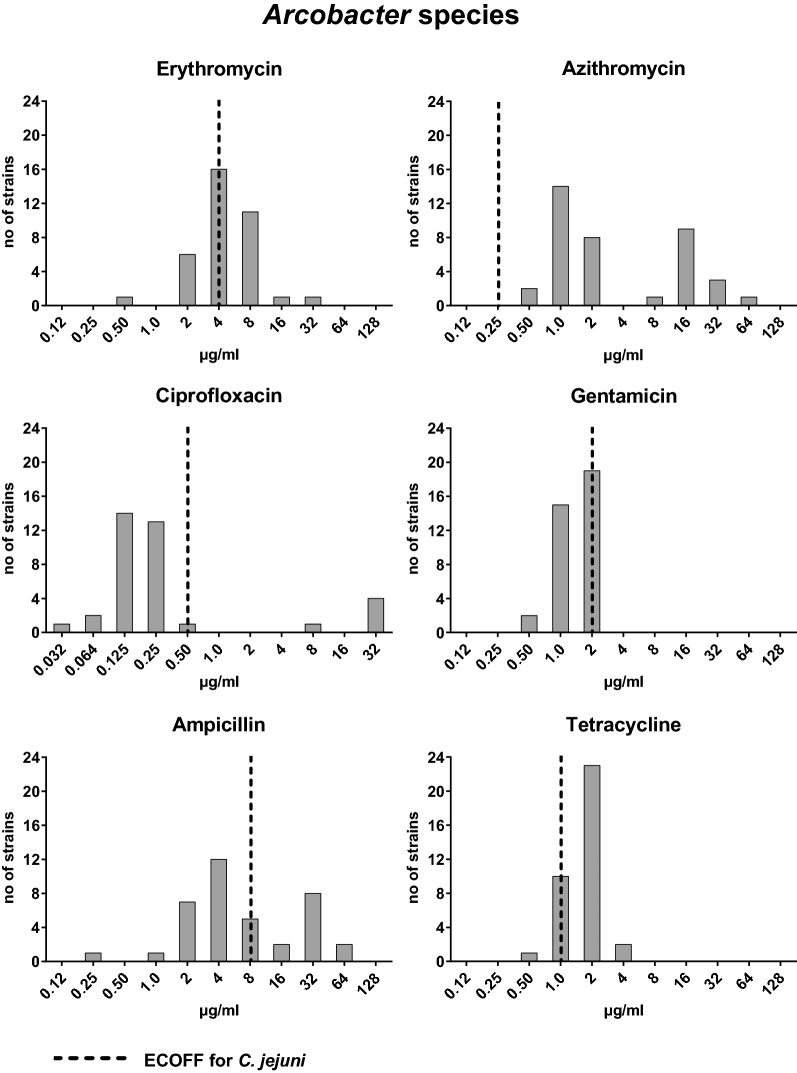
MIC distribution of *Arcobacter* spp. originating from human stool samples for six antimicrobial agents. The MICs determined by gradient strip method were adjusted upwards to the next upper two-fold dilution. Black broken lines: epidemiological cut-offs (ECOFFs) for *C. jejuni*

For antimicrobial susceptibility testing of human *Arcobacter* isolates, six antibiotics were selected. Overall, our results revealed normally distributed minimal inhibitory concentrations (MICs) among *Arcobacter* spp. for erythromycin, ciprofloxacin, gentamicin and tetracycline, while a bimodal distribution for azithromycin and ampicillin was apparent (Fig. 2). For erythromycin, MICs (ranging from 0.5 to 32.0 µg/ml; Table 2) were distributed around the epidemiological cut-off (ECOFF) for *C. jejuni* (4 µg/ml; Fig. 2), while MICs for azithromycin were distributed above the ECOFF of *C. jejuni* (0.25 µg/ml; Fig. 2), ranging from 0.5 to 64.0 µg/ml (Table 2). Elevated MICs for azithromycin (> 8 µg/ml; Fig. 2) were determined for 50% of *A. butzleri* and 10% of *A. cryaerophilus* isolates (Table 2). The majority of all isolates (86%; Table 2) displayed high susceptibility to ciprofloxacin (MIC ≤ 1 µg/ml; Fig. 2), whereas MIC ≥ 4 µg/ml were determined for 2/24 (8%) of *A. butzleri* and 3/10 (30%) of *A. cryaerophilus* isolates (Table 2). Only MICs below the ECOFF for *C. jejuni* (2 µg/ml) were determined for gentamicin, with no species differences (Fig. 2, Table 2). The MICs for ampicillin were bimodally distributed around the ECOFF of *C.* *jejuni* (8 µg/ml; Fig. 2) and 10/24 (42%) of *A.* *butzleri* and 2/10 (20%) of *A.* *cryaerophilus* isolates displayed elevated MICs (> 8 µg/ml; Fig. 2, Table 2). For tetracycline, MICs of all *Arcobacter* spp. isolates were distributed around the ECOFF for *C.* *jejuni* (1 µg/ml; Fig. 2), whereas the MICs determined for both *A. lanthieri* isolates were distributed within the ranges described for the other two species (Table 2).

**Table 2:** MIC distribution for 24 *A. butzleri*, 10 *A. cryaerophilus* and 2 *A. lanthieri* isolates

Antimicrobial agent	Species	No. of strains with MIC (µg/ml) of:													
		0.032	0.064	0.125	0.25	0.50	1	2	4	8	16	32	(> 32)	64	128
Azithromycin	*A. butzleri*					2	2	7		1	8	3		1	
	*A. cryaerophilus*						8	1			1				
	*A. lanthieri*						2								
Ampicillin	*A. butzleri*				1		1	4	4	4		8		2	
	*A. cryaerophilus*							3	4	1	2				
	*A. lanthieri*						1		1						
Ciprofloxacin^a^	*A. butzleri*	1	2	8	11					1			1		
	*A. cryaerophilus*			4	2	1							3		
	*A. lanthieri*			2											
Gentamicin	*A. butzleri*						8	16							
	*A. cryaerophilus*					2	5	3							
	*A. lanthieri*						2								
Erythromycin	*A. butzleri*					1		2	8	11	1	1			
	*A. cryaerophilus*							4	6						
	*A. lanthieri*														
Tetracycline	*A. butzleri*						5	17	2						
	*A. cryaerophilus*					1	4	5							
	*A. lanthieri*						1	1							

^a^The maximum concentration tested by the ciprofloxacin gradient strip was 32 µg/ml. MIC > 32 µg/ml indicate no growth inhibition

## Discussion

### *Arcobacter* prevalence in human stool samples

This is the first prospective study addressing the prevalence of *Arcobacter* in stool samples from outpatients and hospitalized patients in Germany by applying an *Arcobacter*-specific detection method. Overall, *Arcobacter* spp. were isolated from 36 out of a total of 4636 (0.77%) examined specimens. This isolation rate is in concordance with studies from New Zealand and Belgium, where *Arcobacter* spp. were detected in 0.9% (12/1380) and 1.31% (89/6774) of human diarrheal fecal samples respectively [10, 20], whereas slightly different prevelances (as low as 0.2 or up to 3.6%) were found in other studies from Belgium, Turkey, Portugal, India and Chile [12, 21–24]. These differences could be attributed to various factors, such as patient populations, geographical aspects, examined sample sizes, and in particular, to the different microbiological methods applied. The impact of the detection method has been demonstrated in several studies [25–28]. The authors each compared different cultural isolation strategies with varying incubation and medium conditions revealing differences in *Arcobacter* isolation frequency ranging from 7% to 36%. Notably, our study revealed a higher *Arcobacter* prevalence in an analyzed subgroup by using *Arcobacter*-specific enrichment (0.97%) than determined by non-specific methods used in the three routine laboratories (0.49%). Future studies should address whether patients with *Arcobacter* spp. at low quantities that can only be detected by applying specific enrichment methods differ clinically from those patients in whom the pathogen is easily detected within the routine culture-based procedures.

Furthermore, we determined a higher *Arcobacter* prevalence in stool samples of outpatients than of hospitalized patients (i.e., 0.85% (33/3884) and 0.40% (3/752), respectively). Thus, in most patients, *Arcobacter* spp. most likely do not cause serious infections requiring hospitalization. Likewise, in a previous German study, patients who were hospitalized for severe gastroenteritis (n = 104) were found to be positive mainly for norovirus or *Campylobacter* spp.; in contrast, no *Arcobacter* was isolated by using routine diagnostics [29].

Among the 36 *Arcobacter* isolates obtained in our study, *A.* *butzleri* was the most prevalent species (n = 24) followed by *A.* *cryaerophilus* (n = 10), which is in line with other studies [10, 21, 24]. In addition, to best of our knowledge, this is the first report of *A.* *lanthieri* isolation from human specimens (n = 2) which might point towards its role as gastrointestinal pathogen. However, the applied selective enrichment media as well as the multiplex PCR are validated for the detection of the three species *A. butzleri*, *A. cryaerophilus* and *A. skirrowii* only, and could therefore bias the result according to species diversity [16, 18].

Overall, in the analyzed subgroup *Arcobacter* spp. were the second most frequently isolated pathogens (0.97%) after *Campylobacter* spp. (4.39%), followed by *Salmonella enterica* (0.75%). Our results are supported by a previous study demonstrating *Arcobacter* spp. as fourth most commonly isolated pathogens from diarrheal patients (1.31%), after *Campylobacter* spp. (5.61%), *Salmonella* spp. (2.04%) and *C. difficile* (1.61%), albeit prevalences of the enteropathogens were higher than in our study [10].

## Antimicrobial susceptibility

Data regarding antimicrobial susceptibilities of *Arcobacter* spp. are scarce, mainly due to missing standardized protocols and defined breakpoints, which makes it difficult to interpret results and to define antimicrobial resistance. In previous studies, MIC results have been compared with breakpoints for *Enterobacteriaceae* or *Staphylococcus* spp. as defined by the Clinical Laboratory Standards Institute (CLSI), with breakpoints for *Campylobacter* as defined by the U.S. National Resistance Monitoring System criteria or with EUCAST breakpoints for *Enterobacteriaceae, Campylobacter* or non-species related breakpoints [5, 30, 31]. In our study, we compared the MICs with ECOFFs defined by EUCAST for *C. jejuni* [32]. For ciprofloxacin, gentamicin and ampicillin the *C. jejuni* ECOFFs appear to apply for *Arcobacter* as well as previously proposed by Riesenberg et al. [33]. However, our data suggest that *Arcobacter* ECOFFs for erythromycin, tetracycline and azithromycin may be higher than those of *C. jejuni*. All of our isolates displayed MICs for azithromycin above the ECOFF of *C. jejuni* (0.25 µg/ml), which, however, is comparable with data from a Belgian study [34]. Although erythromycin and azithromycin are both macrolides, the bimodal distribution for azithromycin but not for erythromycin was remarkable. Van den Abeele et al. have also detected MICs > 8 µg/ml for azithromycin in 50% of *A. butzleri* isolates, which is in line with our results [34]. Likewise, other studies revealed elevated MICs for azithromycin in up to 95% of *A. butzleri* and in 20% of *A. cryaerophilus* strains isolated from poultry products [30, 35]. Similar to our results, other studies on antimicrobial susceptibility revealed also low MICs for *Arcobacter* spp. to erythromycin whereas some studies reported resistance rates up to 62% [5, 36, 37]. In contrast to our study, those studies used disc diffusion assays with 15 µg/disc and applied resistance criteria for *Enterobacteriaceae* according to CLSI 2010. In *Campylobacter,* there is usually cross-resistance between azithromycin and erythromycin. Single isolates, however, may display susceptibility to erythromycin and resistance to azithromycin, and whole genome sequencing analysis revealed an amino acid substitution in ribosomal protein L22 (leading to azithromycin resistance), but no mutations in the 23S rRNA gene, which explains the susceptibility to erythromycin [38]. Further analyses are needed to determine the genomic background being responsible for the divergent MIC distributions observed by us for *Arcobacter* spp.

As mentioned before, 86% of the investigated *Arcobacter* isolates showed low MICs for ciprofloxacin ranging from 0.032–0.50 µg/ml, which is further supported by a recent study reporting ciprofloxacin susceptibility for all tested *Arcobacter butzleri* isolates [36]. In contrast, clinical *Campylobacter* isolates displayed high resistance rates (MICs ≥ 4 µg/ml) ranging from 45 to 71.4% [39, 40]. Notably, we found elevated MICs for ciprofloxacin predominantly in *A. cryaerophilus* strains similar to a Belgian study [34]. Thus, ciprofloxacin might be the drug of choice, if antibiotic treatment of *A. butzleri*-infection is required.

In accordance with our data, only low resistance rates from 0–4% of *Arcobacter* spp. to gentamicin have been reported before [36]. Similarly, susceptibility to tetracycline might be common, although one recent study from retail food in Portugal demonstrated high resistance (95%) in *A. butzleri* [5, 41]. Furthermore, 42% of our *A. butzleri* isolates displayed high MICs for ampicillin (24–64 µg/ml), which is similar to previous studies where 50 to 100% isolates with high ampicillin MICs have been shown [20, 22, 31, 34].

## Conclusions

In summary, *Arcobacter* spp. were not rare in our study and could be isolated more often from outpatients than from hospitalized patients. Furthermore, *A.* *lanthieri* was identified in fecal samples from human patients for the first time. Results from antimicrobial susceptibility testing indicate that *Arcobacter* spp. might be more susceptible to fluoroquinolones than to macrolides, particularly azithromycin. Future studies should provide reliable risk assessments of *Arcobacter* infections in humans.

## Methods

### Isolation of *Arcobacter* spp

During a 13-month survey (from October 2017 until October 2018) 4636 stool samples were collected at three microbiological diagnostic laboratories in Berlin, Germany. Only stool samples submitted for the detection of bacterial enteropathogens were included. Given that samples were pseudonymized before performance, no detailed patient information were available. Samples were stored up to 1 week at 4 °C by the diagnostic laboratories until *Arcobacter* specific isolation procedures were performed in our laboratories.

For detection of *Arcobacter* spp., isolation was carried out using selective enrichment media according to a study done by van Driessche et al. [16]. All incubation steps were performed at 30 °C under microaerobic conditions unless stated differently. Briefly, 1 g of stool samples was diluted at 1:10 with *Arcobacter* broth (Oxoid, Wesel, Germany) (24 g/l) containing 5% lysed horse blood, 5´-fluorouracil (100 mg/l), amphotericin B (10 mg/L), novobiocin (32 mg/l), cefoperazone (16 mg/l) and trimethoprim (64 mg/l) (Sigma-Aldrich, Taufkirchen, Germany). The samples were mixed thoroughly, and incubated for 72 h. Samples were then plated onto *Arcobacter* selective plates (as described above except lysed horse blood) and incubated for 48 h. Suspect colonies (i.e., small round white or grey colonies) were transferred onto Mueller–Hinton agar plates (Oxoid) supplemented with 5% sheep blood (MHB) and incubated for 48 h.

### PCR analyses

The genomic DNA of these isolates was extracted by using a modified chelex-based method described by Karadas et al. [17]. Briefly, a small amount of colony material was washed in 250 µl TE buffer (1 mM Tris/HCL, pH 8.0, 100 µM EDTA; Roth, Karlsruhe, Germany) and pelleted by centrifugation at 16,000×*g* for 6 min. Pellets were resuspended in 250 µl 5% Chelex (BioRad, Munich, Germany) followed by incubation at 56 °C for 1 h and subsequently at 95 °C for 10 min. After centrifugation at 16,000x*g* for 5 min, 100 µl of the supernatant were stored at 4 °C or directly used to identify the isolates by multiplex PCR according to Houf et al. [18]. Briefly, PCR reaction mixture contained 1x PCR buffer (Qiagen, Venlo, Netherlands), 2.8 mM MgCl_2_ (Qiagen), 0.2 mM of each deoxynucleoside triphosphate (dNTP) (Thermo Fisher Scientific, Waltham, USA), 0.75 U *Taq* polymerase (Qiagen), 1 µM of each primer ARCO R, BUTZ F, CRY 1, and CRY 2 and 0.5 µM of primer SKIR F, and 2 µl template DNA in a total reaction volume of 25 µl. PCR samples were subjected to an initial denaturation step at 94 °C for 5 min, followed by 32 amplification cycles, consisting of denaturation at 94 °C for 45 s, annealing at 61 °C for 45 s and elongation at 72 °C for 30 s, and subsequently 5 min at 72 °C for final extension. DNA of *A.* *butzleri* (CCUG 30485), *A.* *cryaerophilus* (DSM 7289) and *A.* *skirrowii* (CCUG 10374) were used as control. Amplified products were separated using gel electrophoresis and visualized under UV light by GRgreen staining.

For verification at species level, all positive isolates were analyzed by *rpoB* sequencing according to a study done by Korczak et al. [19]. Briefly, a 50 µl PCR-mixture contained 4 µl template DNA, 1x PCR buffer, 2.5 mM MgCl_2_, 0.2 mM of each dNTP, 1 U *Taq* polymerase and 0.4 µM of each primer CamrpoB-L and RpoB-R. PCR reaction conditions were 95 °C for 3 min followed by 35 cycles of 94 °C for 30 s, 54 °C for 30 s and 72 °C for 30 s and subsequently a final extension step at 72 °C for 7 min. Amplified products were separated using gel electrophoresis and visualized under UV light by GRgreen staining. Amplicons were purified using GeneJET PCR Purification Kit (Thermo Fisher Scientific) according to the manufacturer’s instructions and sequenced by GATC (Eurofins GATC Biotech, Konstanz, Germany). Species were identified by comparing the *rpoB* sequences with BLAST database (NCBI).

### Antimicrobial susceptibility testing

Susceptibility testing of *Arcobacter* spp. isolates to azithromycin, ampicillin, ciprofloxacin, gentamycin, erythromycin and tetracycline was performed using the gradient strip diffusion method (*E*-test^TM^, bioMérieux, Nürtingen, Germany). Briefly, *Arcobacter* isolates grown on MHB agar plates (30 °C, microaerobic, 48 h) were precultured overnight in brucella broth (BB; 30 °C, microaerophilic) to receive an inoculum of approximately 1 x 10^8^ colony forming units (CFU) per ml. *Escherichia coli* ATCC 25922 was used as control and cultured likewise, but at 37 °C and in aerobic atmosphere. For testing the slower growing *A. cryaerophilus* isolates, three overnight cultures per isolate were pooled (6 ml), centrifuged, and the pellets resuspended in 600 µl BB in order to receive similar inoculum concentrations. MHB agar plates were inoculated with 100 µl of preculture and incubated after application of gradient strips at 30 °C for 48 h under microaerobic conditions (37 °C and aerobic for *E. coli*).

### Statistical analysis

For calculating significant differences in prevalences of *Arcobacter* in outpatients and hospitalized patients, the Chi squared test and the Fisher’s exact test were performed using GraphPad Prism (version 5.04; GraphPad Software, Inc., La Jolla, US). Differences were considered significant at values of P < 0.05.

## Data Availability

All data generated or analysed during this study are included in this published article.
